# An analysis of drug resistance among people living with HIV/AIDS in Shanghai, China

**DOI:** 10.1371/journal.pone.0165110

**Published:** 2017-02-10

**Authors:** Fengdi Zhang, Li Liu, Meiyan Sun, Jianjun Sun, Hongzhou Lu

**Affiliations:** 1 Department of Infectious Disease, Shanghai Public Health Clinical Center, Fudan University, Shanghai, China; 2 Department of Infectious Disease, Huashan Hospital Affiliated to Fudan University, Shanghai, China; 3 Department of Internal Medicine, Shanghai Medical College, Fudan University, Shanghai, China; UCL, UNITED KINGDOM

## Abstract

**Background:**

Understanding the mechanisms of drug resistance can facilitate better management of antiretroviral therapy, helping to prevent transmission and decrease the morbidity and mortality of people living with HIV/AIDS. However, there is little data about transmitted drug resistance and acquired drug resistance for HIV/AIDS patients in Shanghai.

**Methods:**

A retrospective cohort study of HIV-infected patients who visited the Department of Infectious Disease from June 2008 to June 2015 was conducted in Shanghai, China. Logistic regression analysis was performed to analyze risk factors for drug resistance among HIV-infected people with virological failure. The related collected factors included patient age, gender, marital status, infection route, baseline CD4 count, antiretroviral therapy regimens, time between HIV diagnosis and initiating antiretroviral therapy. Factors with p<0.1 in the univariate logistic regression test were analyzed by multivariate logistic regression test.

**Results:**

There were 575 subjects selected for this study and 369 participated in this research. For the antiretroviral therapy drugs, the rates of transmitted drug resistance and acquired drug resistance were significantly different. The non-nucleoside reverse transcriptase inhibitor (NNRTI) had the highest drug resistance rate (transmitted drug resistance, 10.9%; acquired drug resistance, 53.3%) and protease inhibitors (PIs) had the lowest drug resistance rate (transmitted drug resistance, 1.7%; acquired drug resistance, 2.7%). Logistic regression analysis found no factors that were related to drug resistance except marital status (married status for tenofovir: odds ratio = 6.345, 95% confidence interval = 1.553–25.921, P = 0.010) and the time span between HIV diagnosis and initiating antiretroviral therapy (≤6M for stavudine: odds ratio = 0.271, 95% confidence interval = 0.086–0.850, P = 0.025; ≤6M for didanosine: odds ratio = 0.284, 95% confidence interval = 0.096–0.842, P = 0.023; ≤6M for tenofovir: odds ratio = 0.079, 95% confidence interval = 0.018–0.350,P<0.001).

**Conclusion:**

NNRTI had a higher DR rate compared with nucleoside reverse transcriptase inhibitor (NRTI) and PIs, consequently, LPV/r was a reasonable choice for patients with NNRTI drugs resistance in China. Only married status and a time span≤6 month between the HIV confirmed date and the time initiating antiretroviral therapy were risk factors for TDF drug resistance. Both baseline HIV-RNA load and resistance test is crucial for TDR diagnosis, and frequent monitoring of HIV-RNA load is crucial for ADR identification and intervention. Treatment adherence still plays a positive role on the outcome of ART.

## Introduction

Combined antiretroviral therapy (cART) has significantly decreased the morbidity and mortality of people living with HIV/AIDS (PLWHA)[1]. It has been nearly two decades since cART emerged as a treatment for human immunodeficiency virus type 1 (HIV-1) infection, but drug resistance (DR) is well documented [2]. Drug resistance can be categorized into transmitted drug resistance (TDR) and acquired drug resistance (ADR), both of which present serious threats to PLWHA. The development of drug resistance and the effect of antiretroviral therapy(ART) drugs on virus resistance should be key considerations in the selection of ART regimens for PLWHA [3]. In some settings, just like the researches of both Hoffmann et al[4] and Gupta et al[5] showed that re-suppression can occur when there is drug resistance. Generally, however, ignoring TDR can result in treatment failure of antiretroviral regimens, and ADR is often associated with virological failure (VF) and can increase the burden of treatment [6].

Many factors can influence the presence of ADR. A low CD4 count and high HIV-RNA virus load (V-L) at baseline can contribute to a high ADR rate [3,7]. In addition to the influence of ART drugs and HIV virus, ADR is also affected by adherence to ART[8,9].

Distinct features on the control and management of the HIV epidemic in China should be a primary concern. Our team’s previous study summarized drug resistance characteristics of NRTI and NNRTI, but related data in recent years is scarce. With the development of ART drugs in China, a change had taken place in the types of drugs resistance in recent clinical settings [10]. ART regimens vary in different locations, and first-line ART drugs used in lower income countries may not be as advanced as those used in high income countries. In terms of surveillance, baseline HIV-RNA load testing is typically not covered by insurance or other support, although a free HIV-RNA load test is suggested annually after ART initiation according to Chinese policy. The aforementioned factors can all affect drug resistance but little is known about features of drug resistance in China. Therefore, we conducted a retrospective study to analyze drug resistance.

## Materials and methods

### Ethics statement

The study protocol was submitted and approved by the Shanghai Public Health Clinical Center Ethics Committee. The Ethics Committee authorized this study without written informed consent from participants because the study was retrospective and anonymous.

### Study design, subjects and inclusion criteria

This retrospective cohort study included HIV-infected patients who were patients at the Department of Infectious Disease of Shanghai Public Health Clinical Center from June 2008 to June 2015 in Shanghai, China. Subjects had a HIV-RNA virus load higher than 1000 copies/mL and volunteered for drug resistance testing. The WHO stage of participants was assessed at the first visit by clinicians. Regardless of whether or not patients started ART, all were included in this study. ART regimens conformed to the current Guideline of Diagnosis and Treatment of AIDS in China: Zidovudine (AZT) and Lamivudine (3TC) with either Efavirenz(EFV) or Neviripine(NVP); Stavudine(d4T) and 3TC with either EFV or NVP; Tenofovir(TDF) and 3TC with EFV or NVP; and other regimen (3TC)+Abacavir(ABC)+lopinavir/ritonavir (LPV/r), AZT /d4T +3TC+ didanosine(ddI)/ indinavir (IDV), AZT+IDV+EFV, d4T+ddI+NVP.

### Data collection

Patients who were tested for drug resistance were recorded in the Hospital Laboratory Retrieval System (HLRS). This study utilized the HLRS to obtain the list of patients that had been tested. At the same time, this research reviewed patient demographic characteristics including gender, age, infection route, marital status, and HIV confirmed time, as well as disease relevant information including CD4 counts, HIV-RNA load, and ART drug resistance from the Hospital Information System(HIS). See S1 File.

### Measurement of HIV-RNA load, CD4 count and drug resistance

Blood samples for HIV-RNA virus load and CD4+ T-Cell count measurement were analyzed with COBAS TaqMan (Roche, Switzerland) and CYTOMICS-FC500, respectively, at the Shanghai Public Health Clinical Center affiliated with Fudan University. Viral RNA was extracted from 140μl plasma using QIAmp Viral RNA Mini Kit (Qiagen, Germany). Reverse Transcription (RT) and nested Polymerase Chain Reaction (PCR) were used to amplify an approximate 1300bp fragment of the *pol* gene spanning the polymerase domain of PR and RT regions. RT-PCR was mainly conducted with Recombiant RNase Inhibitor (TaKaRa, China) and Reverse Transcriptase M-MLV (TaKaRa, China). Nested PCR was performed with Ex Taq (TaKaRa, China). HIV-1 genotypic and ART drug resistance analysis was performed using the Stanford University HIV Drug Resistance Database (http://hivdb.stanford.edu/).

### Data analysis and statistics

Data analysis was conducted using IBM SPSS version 20.0 (IBM SPSS, Inc., Armonk, NY, USA). Continuous variables were described with mean and standard deviation (SD); categorical variables were described by numbers and percentages. Categorical variables were analyzed with the chi-square test or Fisher’s exact test and continuous variables were analyzed using the t-test. Risk factors for drug resistance were analyzed using the logistic regression test. If factors were determined to be p<0.1 in the univariate logistic regression test, they were then analyzed using a multivariate logistic regression test. All hypothesis testing was two-sided, with a level of α = 0.05.

## Results

### Participant characteristics

**Fig 1:**
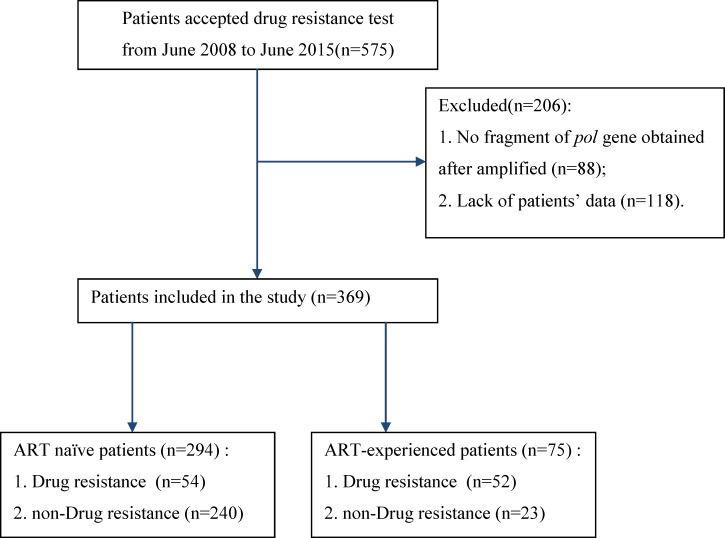
Flow Chart of the Study.

Five hundred and seventy five subjects were considered for inclusion in this study. Based on the exclusion criteria, 206 patients were excluded. The 369 remaining subjects were enrolled in the study. Among these participants, 294 were treatment-naïve patients and 75 were ART-experienced patients, as shown in Fig 1.

For ART naïve patients and ART-experienced patients, there were no differences in demographic characteristics of age, gender, marital status, or infection route between the DR and non-DR groups. CD4 counts were relatively low among participants, with 278 patients (75.3%) below 200cells/μl. In ART naïve patients, more than half the patients (161/294) had a HIV-RNA virus load higher than 4 log10 (>10,000 copies/ml). In ART-experienced patients, no more than 30% (22/75) had HIV-RNA virus load higher than 4 log10.

Among ART naïve patients, most participants (224/294) had drug resistance testing within six months of their HIV confirmation date (HIV CD). Among ART-experienced patients, more than half the participants (38/75) started ART within six months of their HIV CD, while one-third of patients (25/75) had drug resistance testing within a year of starting ART. Among 75 ART-experienced patients, the most used regimens were TDF+3TC+EFV (20/75) and AZT+3TC+EFV (17/75). We analyzed subtypes and found that there were 102 subtype B, 16 subtype C, 164 subtype CRF01_AE, and 12 CRF07_BC among the ART naïve patients. In the ART-experienced patients, there were 38 subtype B, 5 subtype C, 32 subtype CRF01_AE, and 0 subtype CRF07_BC (Table 1).

**Table 1:** The difference of demographic and clinical data in 191 patients with and without drug resistance (DR and non-DR).

Characteristics		ART naïve patients (n = 294)			ART-experienced patients (n = 75)		
		DR(n = 54)	non-DR(n = 240)	p-value	DR(n = 52)	non-DR(n = 23)	p-value
**Age(M±SD)**		37.4±12.3	40.9±14.1	0.069☆	38.5±13.0	43.3±12.3	0.980☆
	≤40(n = 216)	38(70.4)	134(55.8)	0.050	32(61.5)	12(52.2)	0.448
	>40(n = 153)	16(29.6)	106(44.2)		20(38.5)	11(47.8)	
**Gender**							
	Male(n = 335)	48(88.9)	223(92.9)	0.319	43(82.7)	21(91.3)	0.331
	Female(n = 34)	6(11.1)	17(7.1)		9(17.3)	2(8.7)	
**Marital status**							
	Married or live together (n = 207)	29(53.7)	141(58.8)	0.497	27(51.9)	10(43.5)	0.500
	Single, divorced, or widowed (n = 162)	25(46.3)	99(41.2)		25(48.1)	13(56.5)	
**Infection route**							
	Homosexually(n = 331)	49(90.7)	223(92.9)	0.583	41(78.8)	18(78.3)	0.955
	Non-homosexually(n = 38)	5(9.3)	17(7.1)		11(21.2)	5(21.7)	
**CD4 (M±SD)**		118.8±113.4	120.2±130.2	0.367☆	126.8±127.2	137.2±138.5	0.515☆
	≤200(n = 278)	41(75.9)	180(75.0)	0.887	40(76.9)	17(73.9)	0.778
	>200(n = 91)	13(24.1)	60(25.0)		12(23.1)	6(26.1)	
**V-L (Log**_**10**_**)**							
	≤4(n = 186)	27(50.0)	106(44.2)	0.436	37(71.2)	16(69.6)	0.889
	>4(n = 183)	27(50.0)	134(55.8)		15(28.8)	7(30.4)	
**ART regimens**							
	TDF+3TC+EFV(n = 20)	—	—	—	16(30.8)	4(17.4)	0.884
	AZT+3TC+EFV(n = 17)	—	—	—	11(21.2)	6(26.1)	
	AZT+3TC+NVP(n = 10)	—	—	—	7(13.5)	3(13.0)	
	d4T+3TC+EFV(n = 8)	—	—	—	5(9.6)	3(13.0)	
	d4T+3TC+NVP(n = 9)	—	—	—	6(11.5)	3(13.0)	
	Other regimen(n = 11)	—	—	—	7(13.5)	4(17.4)	
**(HIV) CD to DR testing**							
	≤6M(n = 224)	36(66.7)	188(78.3)	0.069	—	—	—
	>6M(n = 70)	18(33.3)	52(21.7)		—	—	—
**(HIV) CD to ART**							
	≤6M(n = 38)	—	—	—	29(55.8)	9(39.1)	0.184
	>6M(n = 37)	—	—	—	23(44.2)	14(60.9)	
**ART to DR testing**							
	≤12M(n = 25)	—	—	—	18(34.6)	7(30.4)	0.723
	>12M(n = 50)	—	—	—	34(65.4)	16(69.6)	

(HIV) CD = HIV confirmed date. Results are shown as n (%) or M±SD.

☆ P values were calculated by t-test. Other values were analyzed by chi-square test or Fisher’s exact test.

### Analysis of Drug Resistance (DR)

**Fig 2:**
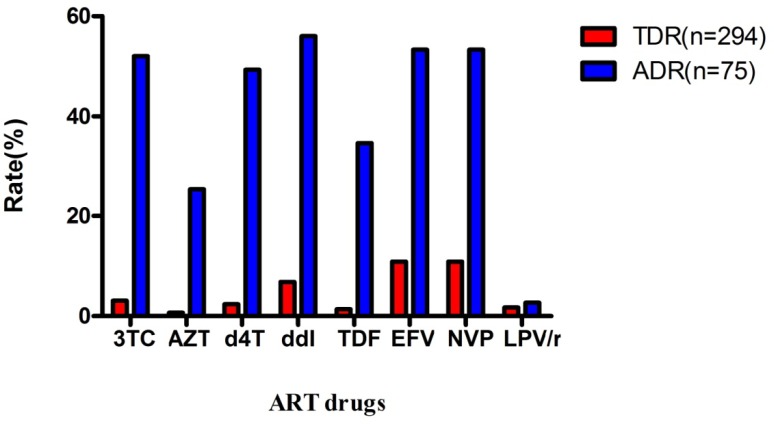
TDR and ADR of ART drugs most frequently used in China.

TDR and ADR rates were significantly different among ART drugs. Understandably, PIs (LPV/r) had the lowest DR rate: 1.7% TDR rate and 2.67% ADR rate. NNRTI (EFV and NVP) exhibited the highest DR rate: 10.88% TDR rate and 53.33% ADR rate for both EFV and NVP. AZT had the lowest TDR rate (0.68%) among first-line commonly used ART drugs, but its ADR rate was not comparably low (25.33%). As expected, NRTI had a moderate DR rate compared with NNRTI and PIs (TDR: TDF 1.36%, d4T 2.38%, 3TC 3.06%; ADR: TDF 34.67%, d4T 49.33%, 3TC 52%). See Fig 2.

**Fig 3:**
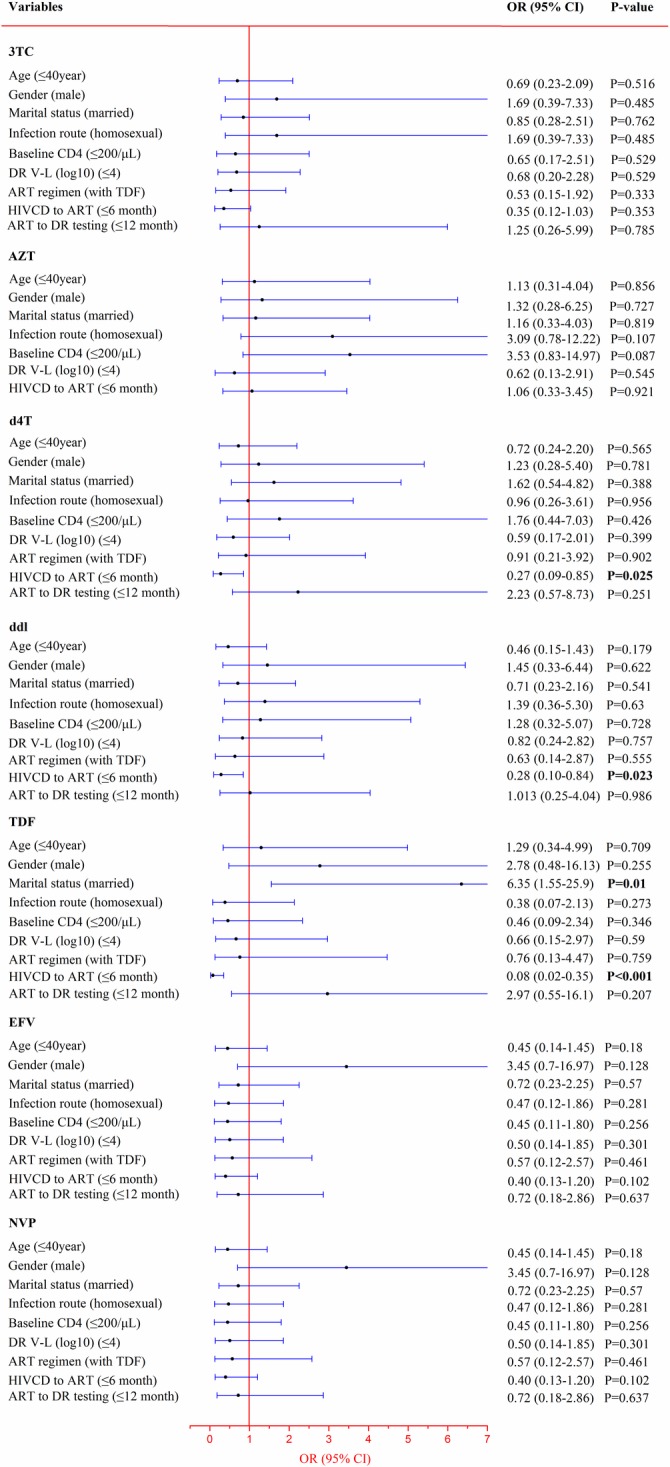
Logistic Regression Analysis for All Drug Resistance Factors in the ART-experienced Group. Few participants used TDF and few participants had ≤12M between ART to DR testing, so these were not included in the logistic regression analysis for AZT drug resistance. Significant results are shown in bold.

To determine the factors associated with drug resistance, logistic regression analysis was conducted. See Fig 3. Of those who were resistant to AZT, only 1 patient used the TDF/3TC/EFV regimen and 2 patients had drug resistance testing within a year of starting ART, so they were excluded from logistic regression analysis. Analysis revealed no relation of factors to drug resistance except for marital status and the time span between HIV CD and starting ART. Married status was a risk factor for TDF drug resistance. Initiating ART within six months of HIV CD was also a risk factor for d4T, ddI, and TDF drug resistance. According to multivariate logistic regression analysis based on the univariate logistic regression, we found that initiating ART within six months of the HIV CD was not a risk factor for d4T drug resistance (OR = 0.496, 95%CI = 0.198–1.244, P = 0.135), but it was the risk factor for ddI (OR = 0.278, 95%CI = 0.106–0.726, P = 0.009) and TDF (OR = 0.123, 95%CI = 0.037–0.409, P = 0.001). Married status was still a risk factor for TDF drug resistance (OR = 4.103, 95%CI = 1.284–13.104, P = 0.017).

### Drug Resistance (DR) and adherence

The hospital provided optimal adherence support and education but a few subjects had unsatisfying adherence for different reasons, see S2 File.

## Discussion

This study identified 54 patients with TDR among 294 ART naïve patients and 52 patients with ADR among 75 ART-experienced patients. For both ART naïve and ART-experienced, participant characteristics between the DR and non-DR groups were comparable. Different HIV-1 subtypes can affect the presence of drug resistance [11,12]. In China, subtype CRF_01AE was previously reported as the most common among people with HIV infection [13]. Consistently, this research showed that subtype CRF_01AE comprised the highest percentage in PLWHA (53.1%) and subtype B accounted for a considerable proportion (37.9%). Subtype CRF_01AE and B accounted for 12.2% and 4.4%, respectively, of DR patients among the ART naïve group. For the ART experienced group, the corresponding proportion was 32.0%. It is noted that in the ART naïve group, the proportion of DR patients with subtype CRF_01AE was nearly three times that of those with subtype B. In contrast, in the ART experienced group, the proportion was approximately the same.

Zidovudine (AZT) was the first nucleoside reverse transcriptase inhibitor for HIV-1 infections [14], and many countries and regions, especially low-income areas, still use it for HIV treatment. Some participants in this study were still using AZT, and this drug performed optimally in drug resistance (0.68% TDR rate and 25.33% ADR rate). The 2015 Guideline for HIV/AIDS Treatment in China did not recommend AZT as a first-line ART drug because of side effects (severe myelo-suppression) and difficulty in compliance (take orally twice a day).

EFV and NVP, as NNRTIs, had the highest drug resistance rate, with 10.88% in the ART-naïve group and 53.33% in the ART-experienced group. NVP is no longer recommended as a first-line ART drug due to its severe adverse effects (hepatotoxicity and allergic reaction) and medication complexity (induction period for reducing side effects) [15]. Similarly, EFV showed a high drug resistance rate and is accompanied by some side effects (central nervous system syndrome) [16]. However, EFV had distinctive features in clinical settings and was one of the most frequently used antiretroviral medicine in ART due to its high potency [17–19]. Many studies showed ART regimens containing EFV had the greatest viral suppression rates in the treatment of naïve HIV patients [20], and the 2015 Guideline for HIV/AIDS Treatment in China recommended EFV as the first-line ART drug. Provided financial resources are available, drug resistance surveillance is crucial given the high DR rate of this drug.

To determine the risk factors of ART drug resistance among the HIV positive population with virological failure, logistic regression analysis was conducted. The results showed that initiating ART within six months from HIV determination to ART initiation was a risk factor for drug resistance of TDF and ddI (p<0.05 for both treatments). However, this situation was not associated with the severity of HIV infection, as evidenced by baseline CD4 count>200ul (p>0.05 for all) or HIV-load>4log10 (p>0.05 for all). Thus, it was related to non-pathological factors, e.g. psychological or social problems. Recent studies showed [21] that early treatment can benefit PLWHA; accordingly, early ART was advised based on individualized risk–benefit analysis. NA Margot and Etiebet[3,22] found that regimens with TDF had higher effects and lower DR rates than regimens including d4T. TDF was not included in the 2011 Guideline for HIV/AIDS Treatment of China, but it was included in the 2015 update. Therefore, regimens with TDF were considered to present less risk for drug resistance, but this study does not support that finding. However, married status was found to be a risk factor of TDF DR. Single, divorced, or widowed HIV infectors were less likely to be resistant to TDF (p = 0.010). Although married participants were more restrained in sexual activity than those that were not married, this was likely not the reason for TDF resistance as no other ART drug resistance was related to marital status. Previous studies showed baseline CD4 count was a risk factor of DR [7], but others revealed no significant difference [23,24]. This study did not find an association of baseline CD4 count with DR occurrence. Similarly, previous studies showed varying results regarding the relationship between baseline HIV-RNA load and the presence of DR [3,7]. Though this study did not include participants’ baseline HIV-RNA loads, HIV-RNA load after ART showed no significant difference between the DR and non-DR groups (p>0.05 for all). Previous studies reported that HIV-1 strains with decreased sensitivity to ART drugs also showed decreased replication, indicated by lower HIV-1 virus load [25,26]. Age, gender, and infection route were not significantly different in the DR and non-DR groups, as reported previously [27–29]. In addition, duration of treatment was not related to DR rate. However, the frequency of monitoring HIV-1 virus load was once a year in this study, but viral load is often tested 3 or 4 times a year in high-income regions. Furthermore, the majority of subjects had a follow-up duration of less than three years. Therefore, better results could potentially be obtained through frequent viral load motoring and longer follow-up duration.

Adherence to treatment also plays a crucial role in the presence of DR [28,30]. Many factors can influence PLWHA’s adherence, including age, regimens, level of formal education received, race/ethnicity, smoking, and other factors [31]. Among 52 participants with DR, 11(21.2%) reported experiences of suboptimal adherence. Other patients’ DR may have been related to ART drugs or interaction between the virus and hosts. Two of 23 subjects without DR also reported suboptimal adherence, which may have been related to their short-term ART (no more than two months). None of the 11 people among the DR group had ART less than two months.

Several limitations of this research should be noted. First, this was retrospective research. The baseline HIV-RNA load was not available and the surveillance frequency of HIV-RNA load was relatively low (once a year) due to local government policy. Therefore, only a marginal percentage of patients (294 of nearly 5000) could afford HIV-RNA load and DR tests at the baseline, and infrequent HIV-RNA load resulted in delayed VF exposure (especially DR). In addition, the following conditions were not observed dynamically with DR, which may further support this research.

In conclusion, NNRTI had a higher DR rate compared with NRTI and PIs, and LPV/r was a satisfying substitute for NNRTI based on this study of patients in China. Only marital status and a time span ≤ 6 months between HIV CD and the initiation of antiretroviral therapy were risk factors for TDF drug resistance. For didanosine, the interval ≤6 month between HIV CD and the start of antiretroviral therapy was the only risk factor. Though early ART can increase DR risk, it is still advised through consideration of risk-benefit analysis. A baseline HIV-RNA load and resistance test is crucial for TDR diagnosis and frequent monitoring of HIV-RNA load is crucial for ADR identification after the initiation of ART. ART adherence can also play a positive role on treatment, so HIV healthcare providers should take effective measures to improve PLWHA adherence.

## Supporting information

S1 File Raw data of participants. (XLSX)

S2 File Reason for poor adherence. (DOCX)
