# Supplementary material for: An analysis of drug resistance among people living with HIV/AIDS in Shanghai, China
Source: PLoS One. 2017 Feb 10;12(2):e0165110. doi: 10.1371/journal.pone.0165110 (PMC5302315; doi:10.1371/journal.pone.0165110)
Supplement: S2 File — (DOCX) [file pone.0165110.s002.docx]

|  | **No. of patients** | **Reasons for bad adherence** |
| --- | --- | --- |
| DR  (n=52) | 5 | Irregular medicinal intake or autonomous withdrawal |
|  | 2 | Lack of knowledge about taking NVP correctly |
|  | 1 | Side effects: dizziness |
|  | 1 | Irregular follow-up |
|  | 1 | Loss of vigilance to HIV after delivery |
|  | 1 | Economic reasons |
| non-DR  (n=23) | 1 | Irregular medicinal intake or autonomous withdrawal |
|  | 1 | Side effects: myelo-suppression |
